# Ammonia in Bismuth Subnitrate

**Published:** 1908-05-30

**Authors:** 


### AMMONIA IN BISMUTH SUBNITRATE.

The following note upon bismuth subnitrate and the ammonia contained in many commercial samples of the drug may be of interest. Attention has been drawn to the matter by Mr. J. R. Hill in the "Pharmaceutical Journal," as the result of difficulties that arose in dispensing the following apparently simple prescription:—

℞ Bismuthi subnitratis ... ... ... gr. xv.
Sodii bicarbonatis ... ... ... gr. xx.
Morphinæ hydrochloridi ... ... gr. $\frac{1}{12}$.
Misce. Fiat pulvis. Mitte tales xij.

When the ingredients were mixed together in a glass mortar a very marked evolution of ammonia occurred. At first it was thought that this might have been due to the mortar not having been made absolutely clean after the dispensing of an ammonium carbonate mixture. It was, therefore, washed out thoroughly once more, and the mixing was repeated. Again there was a strong evolution of ammonia. The question was: Whence came this ammonia? A little investigation showed that it was liberated from the bismuth subnitrate when the latter was triturated with the sodium bicarbonate.

The occurrence of ammonia in bismuth subnitrate has been frequently noted on other occasions. The sample used above belonged to that class of commercial subnitrates which consist of a dull white rather heavy powder which, under the microscope, showed a mixture of prismatic crystals and six-sided plates. Another sample consisting of the whiter and bulkier subnitrate of commerce, and exhibiting under the microscope only small prismatic crystals, was found to liberate ammonia also under similar circumstances, but in relatively much smaller quantities; probably it would not have been detected in the ordinary course of dispensing if particular attention were not being paid to the point.

Every one of the commercial samples examined contained ammonia in varying proportions. This would seem to indicate that in the manufacture of the subnitrate some such process as that described in the United States Pharmacopœia of 1870 is employed, namely, that ammonia water is added in the precipitation of the subnitrate so as to avoid loss of bismuth. Even with such a process, however, the subsequent washing with water should remove any perceptible trace of ammonia. It may be that in the manufacture of the drug a solution of ammonium nitrate is used; for it was suggested by Julius Löwe in 1859 that if a solution of 1 in 500 of ammonium nitrate were used instead of plain water for the washing of a bismuth subnitrate precipitate there would be less risk of losing part of the nitric acid.

The occurrence of a sample in which contamination with ammonia was so obvious suggests that the British Pharmacopœia should insist upon a test for the detection of this impurity.

The United States Pharmacopœia requires that 1 decigram of the subnitrate boiled with 5 cubic centimetres of a test solution of potash should give no perceptible odour of ammonia. It is not quite clear whether this test is intended to exclude ammonia, or whether it is merely desired to fix a limit for the amount that may be allowed. The National Dispensatory says it "should give no reaction for ammonia."

The test in question is, however, by no means a delicate one; for on applying it to the sample in question it was practically impossible to detect any odour of ammonia, although trituration with sodium bicarbonate gave this odour at once. When, however, 1 gramme of the bismuth subnitrate was mixed with 2.5 cubic centimetres of a 50-per-cent. solution of potash, the evolution of ammonia was quite distinct, and a moistened red litmus paper held in the vapour was rapidly turned blue. It seems quite clear that that particular sample contained more ammonia than can be good; and, therefore, since the United States' Pharmacopœial test failed to show this, a more stringent test is desirable, such as the one just mentioned.

The other commercial samples examined, though they all contained ammonia, would have passed as complying with the official standard if the United States test had been relied upon; but all of them without exception failed to pass the more stringent test here suggested.

In any case it is important to know that, in prescribing sodium bicarbonate together with commercial bismuth subnitrate in powder form, as in the prescription from which this discussion arose, there is a great liability for the powders to have an ammoniacal smell when they reach the patient.

